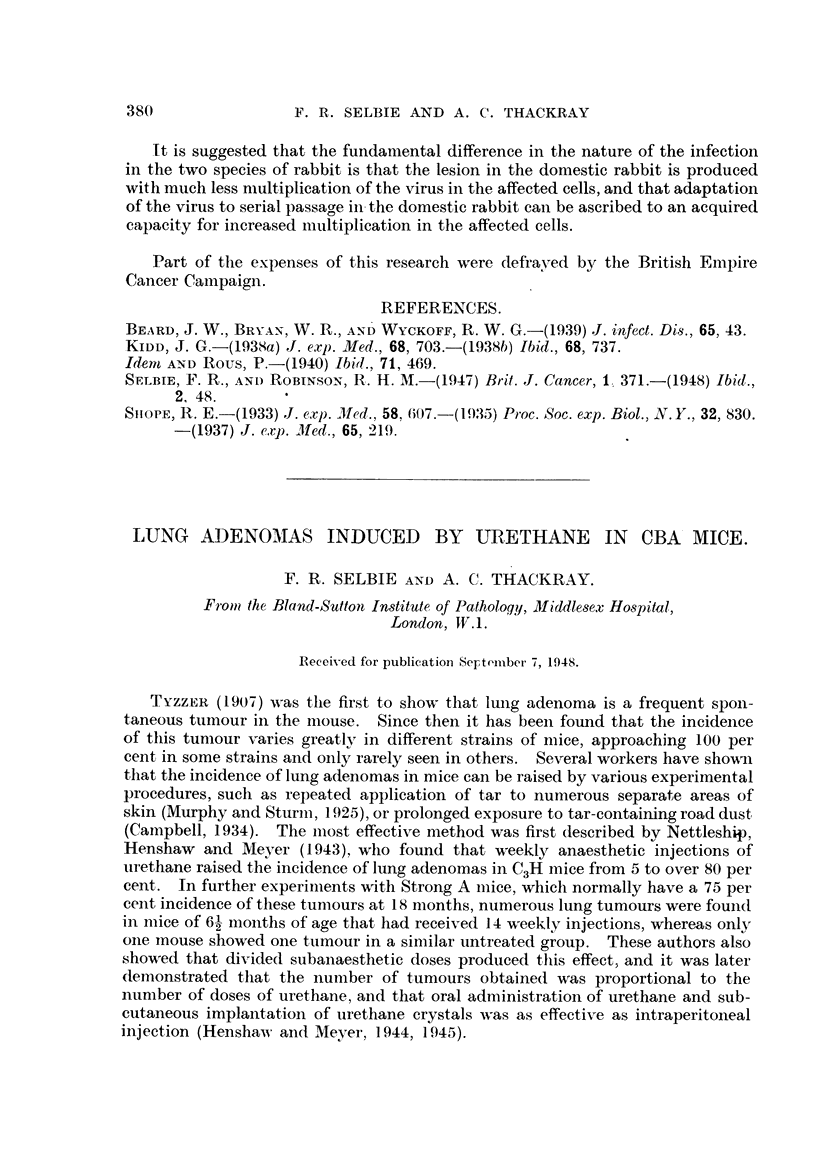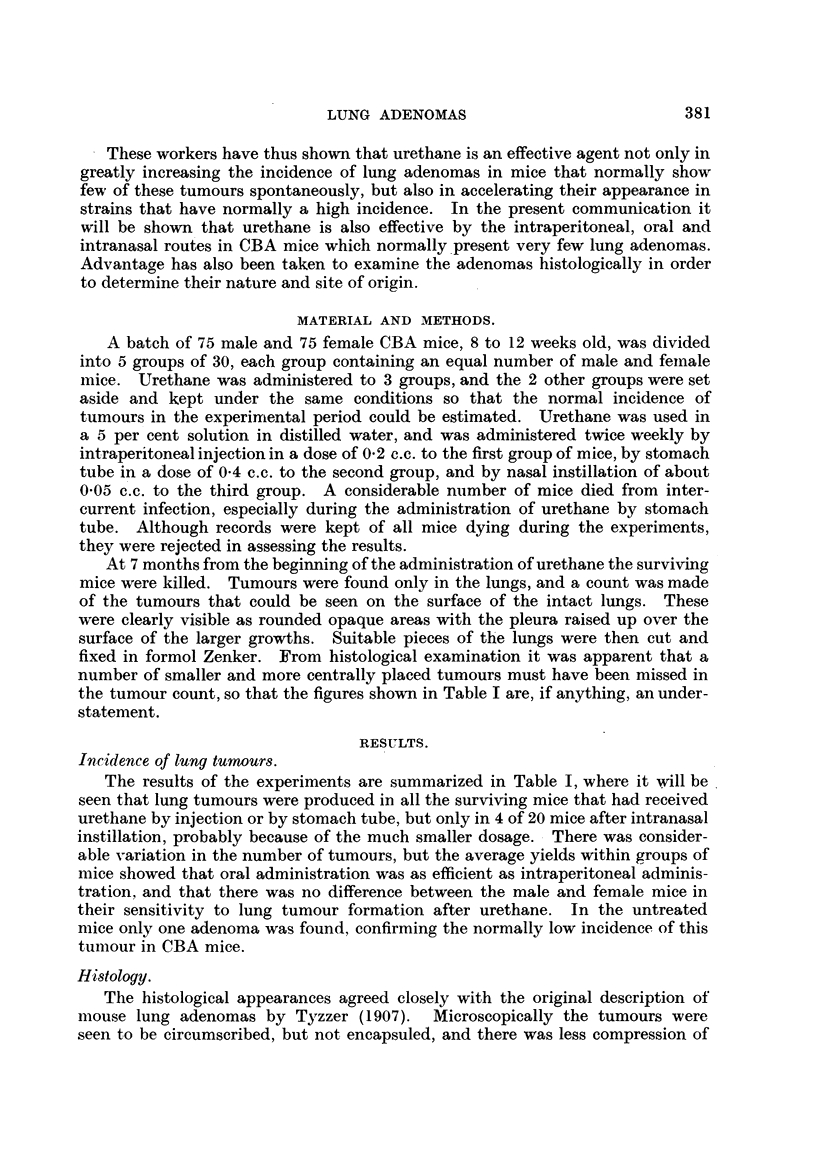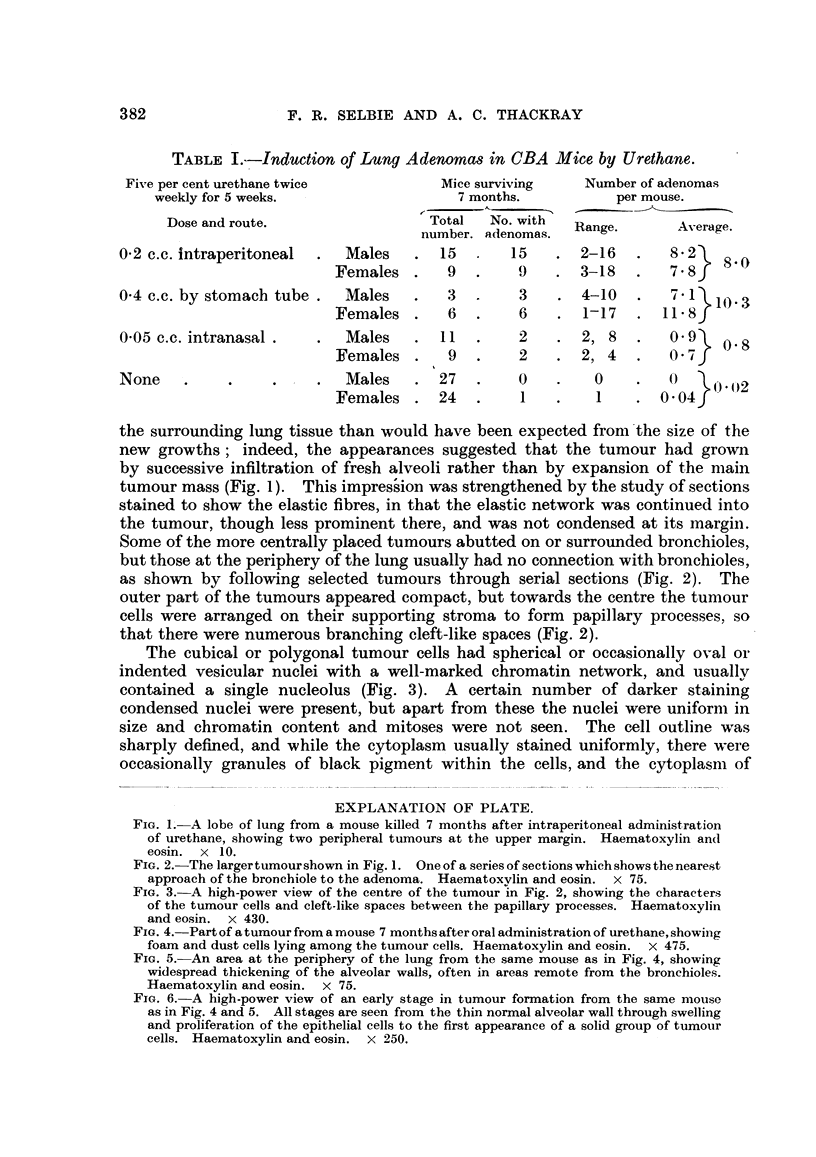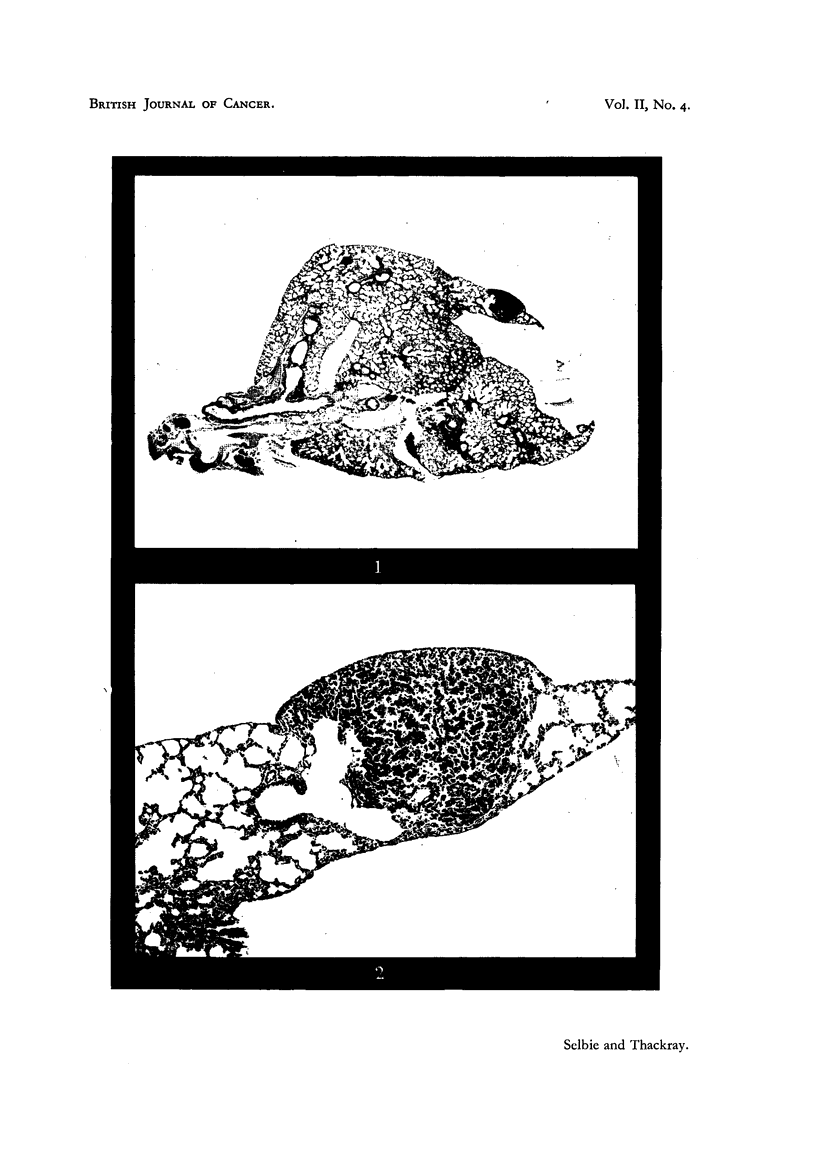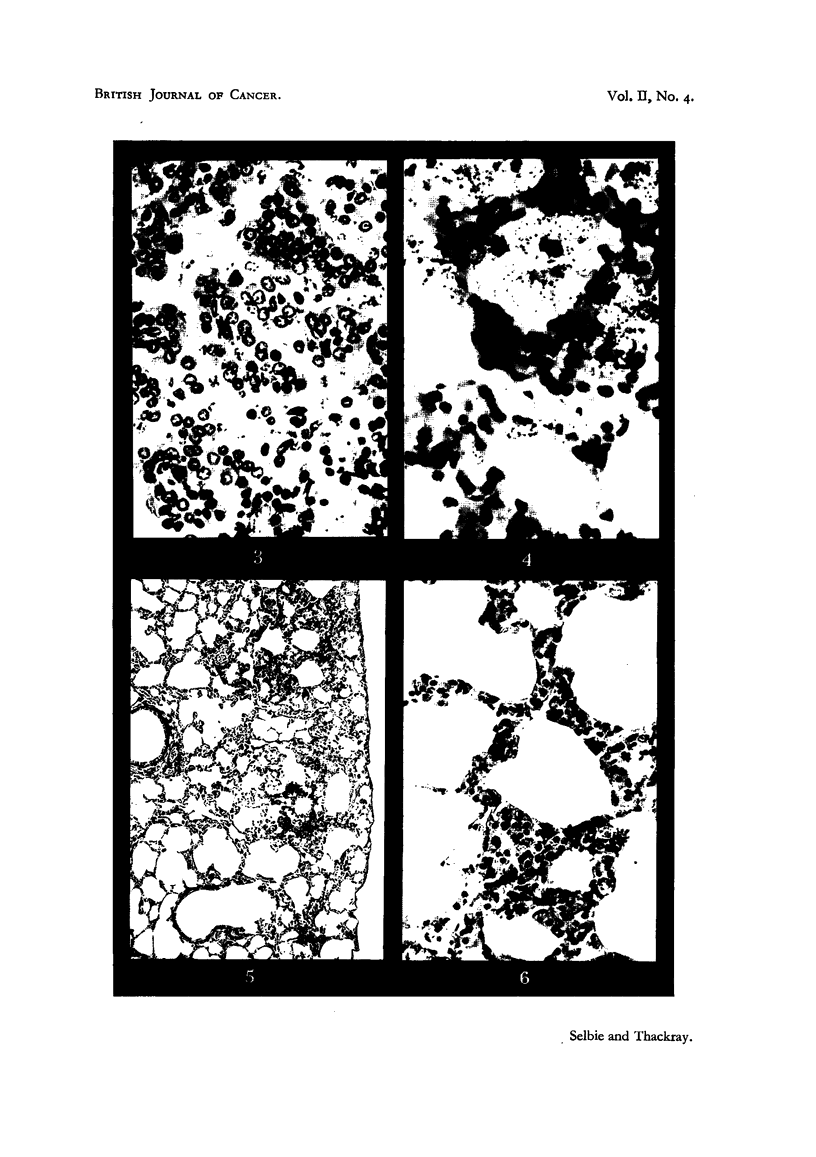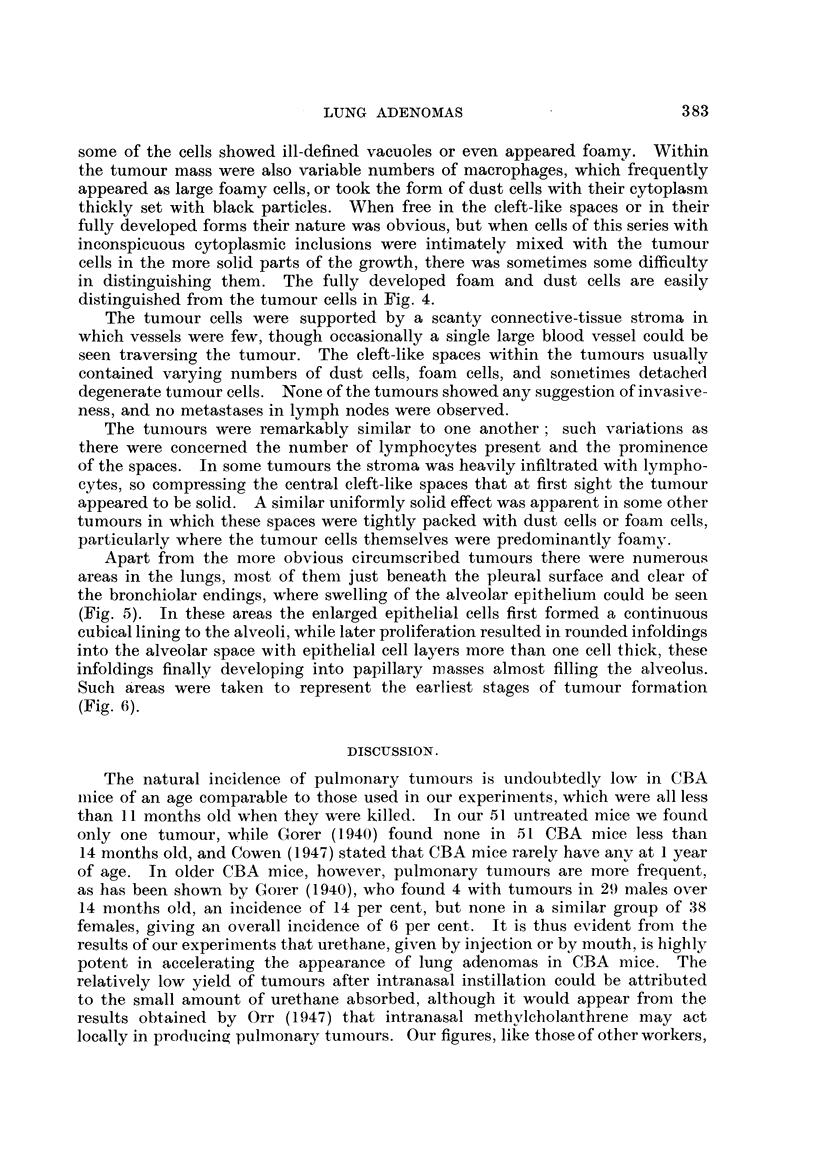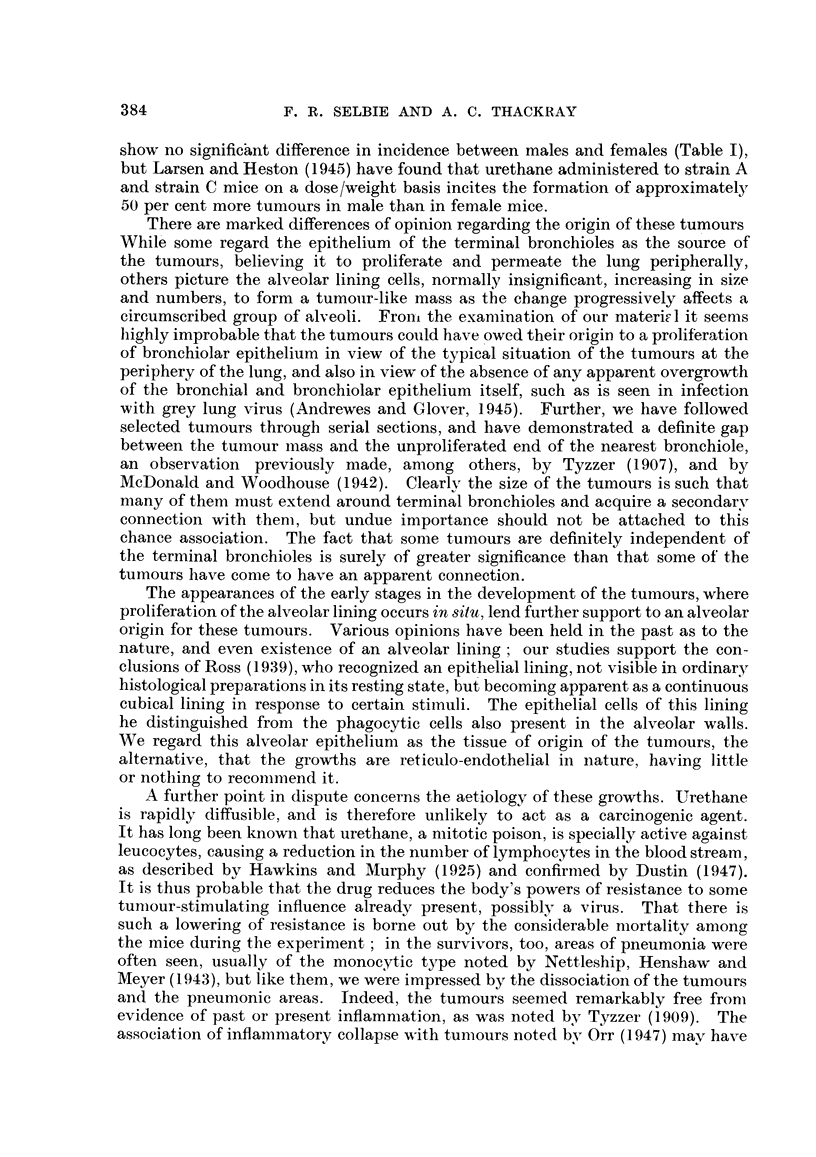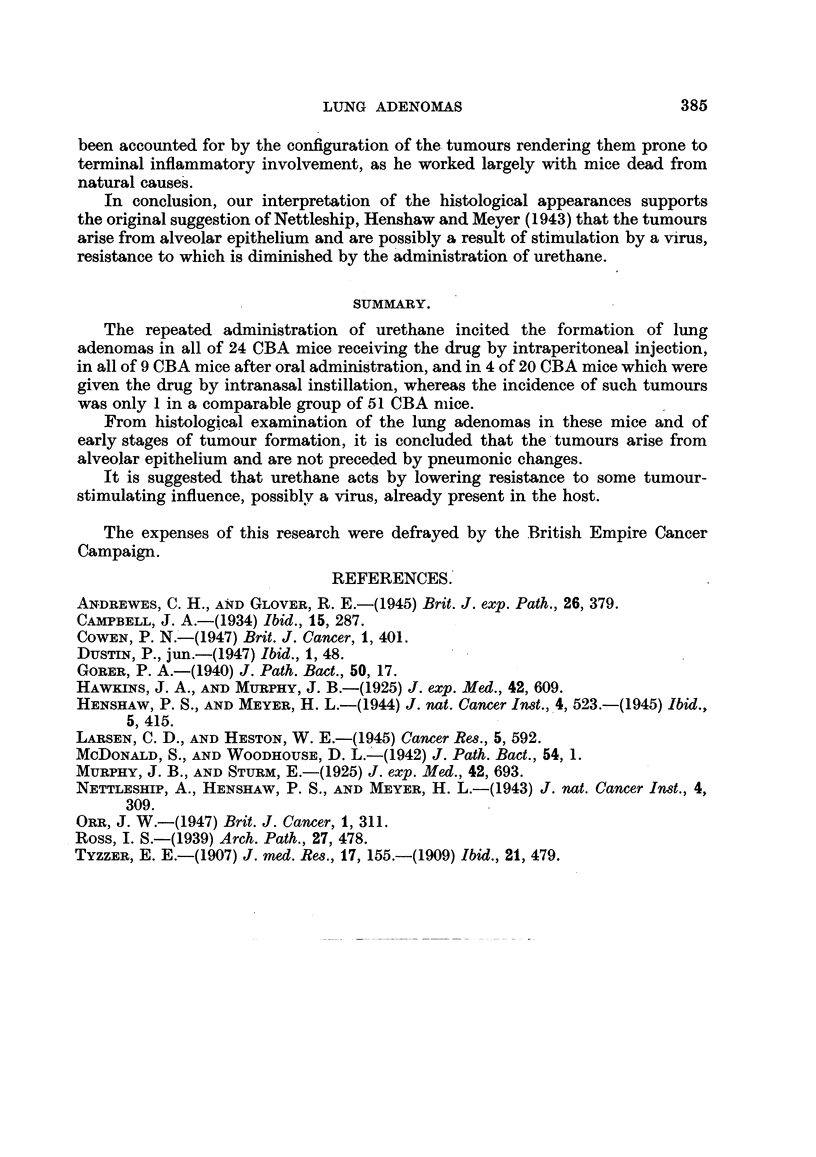# Lung Adenomas Induced by Urethane in CBA Mice

**DOI:** 10.1038/bjc.1948.42

**Published:** 1948-12

**Authors:** F. R. Selbie, A. C. Thackray

## Abstract

**Images:**


### LUNG ADENOMAS INDUCED BY URETHANE IN CBA MICE.

F. R. SELBIE AND A. C. THACKRAY.

*From the Bland-Sutton Institute of Pathology, Middlesex Hospital, London, W.1.*

Received for publication September 7, 1948.

TYZZER (1907) was the first to show that lung adenoma is a frequent spontaneous tumour in the mouse. Since then it has been found that the incidence of this tumour varies greatly in different strains of mice, approaching 100 per cent in some strains and only rarely seen in others. Several workers have shown that the incidence of lung adenomas in mice can be raised by various experimental procedures, such as repeated application of tar to numerous separate areas of skin (Murphy and Sturm, 1925), or prolonged exposure to tar-containing road dust (Campbell, 1934). The most effective method was first described by Nettleship, Henshaw and Meyer (1943), who found that weekly anaesthetic injections of urethane raised the incidence of lung adenomas in $C_3H$ mice from 5 to over 80 per cent. In further experiments with Strong A mice, which normally have a 75 per cent incidence of these tumours at 18 months, numerous lung tumours were found in mice of $6\frac{1}{2}$ months of age that had received 14 weekly injections, whereas only one mouse showed one tumour in a similar untreated group. These authors also showed that divided subanaesthetic doses produced this effect, and it was later demonstrated that the number of tumours obtained was proportional to the number of doses of urethane, and that oral administration of urethane and subcutaneous implantation of urethane crystals was as effective as intraperitoneal injection (Henshaw and Meyer, 1944, 1945).

These workers have thus shown that urethane is an effective agent not only in greatly increasing the incidence of lung adenomas in mice that normally show few of these tumours spontaneously, but also in accelerating their appearance in strains that have normally a high incidence. In the present communication it will be shown that urethane is also effective by the intraperitoneal, oral and intranasal routes in CBA mice which normally present very few lung adenomas. Advantage has also been taken to examine the adenomas histologically in order to determine their nature and site of origin.

### MATERIAL AND METHODS.

A batch of 75 male and 75 female CBA mice, 8 to 12 weeks old, was divided into 5 groups of 30, each group containing an equal number of male and female mice. Urethane was administered to 3 groups, and the 2 other groups were set aside and kept under the same conditions so that the normal incidence of tumours in the experimental period could be estimated. Urethane was used in a 5 per cent solution in distilled water, and was administered twice weekly by intraperitoneal injection in a dose of 0·2 c.c. to the first group of mice, by stomach tube in a dose of 0·4 c.c. to the second group, and by nasal instillation of about 0·05 c.c. to the third group. A considerable number of mice died from intercurrent infection, especially during the administration of urethane by stomach tube. Although records were kept of all mice dying during the experiments, they were rejected in assessing the results.

At 7 months from the beginning of the administration of urethane the surviving mice were killed. Tumours were found only in the lungs, and a count was made of the tumours that could be seen on the surface of the intact lungs. These were clearly visible as rounded opaque areas with the pleura raised up over the surface of the larger growths. Suitable pieces of the lungs were then cut and fixed in formol Zenker. From histological examination it was apparent that a number of smaller and more centrally placed tumours must have been missed in the tumour count, so that the figures shown in Table I are, if anything, an understatement.

### RESULTS.

*Incidence of lung tumours.*

The results of the experiments are summarized in Table I, where it will be seen that lung tumours were produced in all the surviving mice that had received urethane by injection or by stomach tube, but only in 4 of 20 mice after intranasal instillation, probably because of the much smaller dosage. There was considerable variation in the number of tumours, but the average yields within groups of mice showed that oral administration was as efficient as intraperitoneal administration, and that there was no difference between the male and female mice in their sensitivity to lung tumour formation after urethane. In the untreated mice only one adenoma was found, confirming the normally low incidence of this tumour in CBA mice.

*Histology.*

The histological appearances agreed closely with the original description of mouse lung adenomas by Tyzzer (1907). Microscopically the tumours were seen to be circumscribed, but not encapsuled, and there was less compression of

TABLE I.—*Induction of Lung Adenomas in CBA Mice by Urethane.*

| Five per cent urethane twice weekly for 5 weeks. Dose and route. | | Mice surviving 7 months. Total number. | No. with adenomas. | Number of adenomas per mouse. Range. | Average. | |
|---|---|---|---|---|---|---|
| 0·2 c.c. intraperitoneal | Males | 15 | 15 | 2–16 | 8·2 | 8·0 |
| | Females | 9 | 9 | 3–18 | 7·8 | |
| 0·4 c.c. by stomach tube | Males | 3 | 3 | 4–10 | 7·1 | 10·3 |
| | Females | 6 | 6 | 1–17 | 11·8 | |
| 0·05 c.c. intranasal | Males | 11 | 2 | 2, 8 | 0·9 | 0·8 |
| | Females | 9 | 2 | 2, 4 | 0·7 | |
| None | Males | 27 | 0 | 0 | 0 | 0·02 |
| | Females | 24 | 1 | 1 | 0·04 | |

the surrounding lung tissue than would have been expected from the size of the new growths; indeed, the appearances suggested that the tumour had grown by successive infiltration of fresh alveoli rather than by expansion of the main tumour mass (Fig. 1). This impression was strengthened by the study of sections stained to show the elastic fibres, in that the elastic network was continued into the tumour, though less prominent there, and was not condensed at its margin. Some of the more centrally placed tumours abutted on or surrounded bronchioles, but those at the periphery of the lung usually had no connection with bronchioles, as shown by following selected tumours through serial sections (Fig. 2). The outer part of the tumours appeared compact, but towards the centre the tumour cells were arranged on their supporting stroma to form papillary processes, so that there were numerous branching cleft-like spaces (Fig. 2).

The cubical or polygonal tumour cells had spherical or occasionally oval or indented vesicular nuclei with a well-marked chromatin network, and usually contained a single nucleolus (Fig. 3). A certain number of darker staining condensed nuclei were present, but apart from these the nuclei were uniform in size and chromatin content and mitoses were not seen. The cell outline was sharply defined, and while the cytoplasm usually stained uniformly, there were occasionally granules of black pigment within the cells, and the cytoplasm of

EXPLANATION OF PLATE.

FIG. 1.—A lobe of lung from a mouse killed 7 months after intraperitoneal administration of urethane, showing two peripheral tumours at the upper margin. Haematoxylin and eosin. × 10.

FIG. 2.—The larger tumour shown in Fig. 1. One of a series of sections which shows the nearest approach of the bronchiole to the adenoma. Haematoxylin and eosin. × 75.

FIG. 3.—A high-power view of the centre of the tumour in Fig. 2, showing the characters of the tumour cells and cleft-like spaces between the papillary processes. Haematoxylin and eosin. × 430.

FIG. 4.—Part of a tumour from a mouse 7 months after oral administration of urethane, showing foam and dust cells lying among the tumour cells. Haematoxylin and eosin. × 475.

FIG. 5.—An area at the periphery of the lung from the same mouse as in Fig. 4, showing widespread thickening of the alveolar walls, often in areas remote from the bronchioles. Haematoxylin and eosin. × 75.

FIG. 6.—A high-power view of an early stage in tumour formation from the same mouse as in Fig. 4 and 5. All stages are seen from the thin normal alveolar wall through swelling and proliferation of the epithelial cells to the first appearance of a solid group of tumour cells. Haematoxylin and eosin. × 250.

1

2

3 4 5 6

Selbie and Thackray.

some of the cells showed ill-defined vacuoles or even appeared foamy. Within the tumour mass were also variable numbers of macrophages, which frequently appeared as large foamy cells, or took the form of dust cells with their cytoplasm thickly set with black particles. When free in the cleft-like spaces or in their fully developed forms their nature was obvious, but when cells of this series with inconspicuous cytoplasmic inclusions were intimately mixed with the tumour cells in the more solid parts of the growth, there was sometimes some difficulty in distinguishing them. The fully developed foam and dust cells are easily distinguished from the tumour cells in Fig. 4.

The tumour cells were supported by a scanty connective-tissue stroma in which vessels were few, though occasionally a single large blood vessel could be seen traversing the tumour. The cleft-like spaces within the tumours usually contained varying numbers of dust cells, foam cells, and sometimes detached degenerate tumour cells. None of the tumours showed any suggestion of invasiveness, and no metastases in lymph nodes were observed.

The tumours were remarkably similar to one another ; such variations as there were concerned the number of lymphocytes present and the prominence of the spaces. In some tumours the stroma was heavily infiltrated with lymphocytes, so compressing the central cleft-like spaces that at first sight the tumour appeared to be solid. A similar uniformly solid effect was apparent in some other tumours in which these spaces were tightly packed with dust cells or foam cells, particularly where the tumour cells themselves were predominantly foamy.

Apart from the more obvious circumscribed tumours there were numerous areas in the lungs, most of them just beneath the pleural surface and clear of the bronchiolar endings, where swelling of the alveolar epithelium could be seen (Fig. 5). In these areas the enlarged epithelial cells first formed a continuous cubical lining to the alveoli, while later proliferation resulted in rounded infoldings into the alveolar space with epithelial cell layers more than one cell thick, these infoldings finally developing into papillary masses almost filling the alveolus. Such areas were taken to represent the earliest stages of tumour formation (Fig. 6).

### DISCUSSION.

The natural incidence of pulmonary tumours is undoubtedly low in CBA mice of an age comparable to those used in our experiments, which were all less than 11 months old when they were killed. In our 51 untreated mice we found only one tumour, while Gorer (1940) found none in 51 CBA mice less than 14 months old, and Cowen (1947) stated that CBA mice rarely have any at 1 year of age. In older CBA mice, however, pulmonary tumours are more frequent, as has been shown by Gorer (1940), who found 4 with tumours in 29 males over 14 months old, an incidence of 14 per cent, but none in a similar group of 38 females, giving an overall incidence of 6 per cent. It is thus evident from the results of our experiments that urethane, given by injection or by mouth, is highly potent in accelerating the appearance of lung adenomas in CBA mice. The relatively low yield of tumours after intranasal instillation could be attributed to the small amount of urethane absorbed, although it would appear from the results obtained by Orr (1947) that intranasal methylcholanthrene may act locally in producing pulmonary tumours. Our figures, like those of other workers, show no significant difference in incidence between males and females (Table I), but Larsen and Heston (1945) have found that urethane administered to strain A and strain C mice on a dose/weight basis incites the formation of approximately 50 per cent more tumours in male than in female mice.

There are marked differences of opinion regarding the origin of these tumours While some regard the epithelium of the terminal bronchioles as the source of the tumours, believing it to proliferate and permeate the lung peripherally, others picture the alveolar lining cells, normally insignificant, increasing in size and numbers, to form a tumour-like mass as the change progressively affects a circumscribed group of alveoli. From the examination of our material it seems highly improbable that the tumours could have owed their origin to a proliferation of bronchiolar epithelium in view of the typical situation of the tumours at the periphery of the lung, and also in view of the absence of any apparent overgrowth of the bronchial and bronchiolar epithelium itself, such as is seen in infection with grey lung virus (Andrewes and Glover, 1945). Further, we have followed selected tumours through serial sections, and have demonstrated a definite gap between the tumour mass and the unproliferated end of the nearest bronchiole, an observation previously made, among others, by Tyzzer (1907), and by McDonald and Woodhouse (1942). Clearly the size of the tumours is such that many of them must extend around terminal bronchioles and acquire a secondary connection with them, but undue importance should not be attached to this chance association. The fact that some tumours are definitely independent of the terminal bronchioles is surely of greater significance than that some of the tumours have come to have an apparent connection.

The appearances of the early stages in the development of the tumours, where proliferation of the alveolar lining occurs *in situ*, lend further support to an alveolar origin for these tumours. Various opinions have been held in the past as to the nature, and even existence of an alveolar lining ; our studies support the conclusions of Ross (1939), who recognized an epithelial lining, not visible in ordinary histological preparations in its resting state, but becoming apparent as a continuous cubical lining in response to certain stimuli. The epithelial cells of this lining he distinguished from the phagocytic cells also present in the alveolar walls. We regard this alveolar epithelium as the tissue of origin of the tumours, the alternative, that the growths are reticulo-endothelial in nature, having little or nothing to recommend it.

A further point in dispute concerns the aetiology of these growths. Urethane is rapidly diffusible, and is therefore unlikely to act as a carcinogenic agent. It has long been known that urethane, a mitotic poison, is specially active against leucocytes, causing a reduction in the number of lymphocytes in the blood stream, as described by Hawkins and Murphy (1925) and confirmed by Dustin (1947). It is thus probable that the drug reduces the body's powers of resistance to some tumour-stimulating influence already present, possibly a virus. That there is such a lowering of resistance is borne out by the considerable mortality among the mice during the experiment ; in the survivors, too, areas of pneumonia were often seen, usually of the monocytic type noted by Nettleship, Henshaw and Meyer (1943), but like them, we were impressed by the dissociation of the tumours and the pneumonic areas. Indeed, the tumours seemed remarkably free from evidence of past or present inflammation, as was noted by Tyzzer (1909). The association of inflammatory collapse with tumours noted by Orr (1947) may have been accounted for by the configuration of the tumours rendering them prone to terminal inflammatory involvement, as he worked largely with mice dead from natural causes.

In conclusion, our interpretation of the histological appearances supports the original suggestion of Nettleship, Henshaw and Meyer (1943) that the tumours arise from alveolar epithelium and are possibly a result of stimulation by a virus, resistance to which is diminished by the administration of urethane.

### SUMMARY.

The repeated administration of urethane incited the formation of lung adenomas in all of 24 CBA mice receiving the drug by intraperitoneal injection, in all of 9 CBA mice after oral administration, and in 4 of 20 CBA mice which were given the drug by intranasal instillation, whereas the incidence of such tumours was only 1 in a comparable group of 51 CBA mice.

From histological examination of the lung adenomas in these mice and of early stages of tumour formation, it is concluded that the tumours arise from alveolar epithelium and are not preceded by pneumonic changes.

It is suggested that urethane acts by lowering resistance to some tumour-stimulating influence, possibly a virus, already present in the host.

The expenses of this research were defrayed by the British Empire Cancer Campaign.

### REFERENCES.

Andrewes, C. H., and Glover, R. E.—(1945) *Brit. J. exp. Path.*, **26**, 379.
Campbell, J. A.—(1934) *Ibid.*, **15**, 287.
Cowen, P. N.—(1947) *Brit. J. Cancer*, **1**, 401.
Dustin, P., jun.—(1947) *Ibid.*, **1**, 48.
Gorer, P. A.—(1940) *J. Path. Bact.*, **50**, 17.
Hawkins, J. A., and Murphy, J. B.—(1925) *J. exp. Med.*, **42**, 609.
Henshaw, P. S., and Meyer, H. L.—(1944) *J. nat. Cancer Inst.*, **4**, 523.—(1945) *Ibid.*, **5**, 415.
Larsen, C. D., and Heston, W. E.—(1945) *Cancer Res.*, **5**, 592.
McDonald, S., and Woodhouse, D. L.—(1942) *J. Path. Bact.*, **54**, 1.
Murphy, J. B., and Sturm, E.—(1925) *J. exp. Med.*, **42**, 693.
Nettleship, A., Henshaw, P. S., and Meyer, H. L.—(1943) *J. nat. Cancer Inst.*, **4**, 309.
Orr, J. W.—(1947) *Brit. J. Cancer*, **1**, 311.
Ross, I. S.—(1939) *Arch. Path.*, **27**, 478.
Tyzzer, E. E.—(1907) *J. med. Res.*, **17**, 155.—(1909) *Ibid.*, **21**, 479.